# Cardiovascular outcomes among elderly patients with heart failure and coronary artery disease and without atrial fibrillation: a retrospective cohort study

**DOI:** 10.1186/s12872-018-0991-1

**Published:** 2019-01-15

**Authors:** Qi Zhao, Li Wang, Paul A. Kurlansky, Jeff Schein, Onur Baser, Jeffrey S. Berger

**Affiliations:** 1 Janssen Scientific Affairs, LLC, Titusville, NJ USA; 2 STATinMED Research, Plano, TX USA; 3 Columbia University, New York, NY USA; 4 The University of Michigan, Ann Arbor, MI USA; 5 New York University School of Medicine, New York, NY USA

**Keywords:** Coronary artery disease, Myocardial infarction, Ischemic stroke, Mortality

## Abstract

**Background:**

Coronary artery disease accelerates heart failure progression, leading to poor prognosis and a substantial increase in morbidity and mortality. This study was aimed to assess the impact of coronary artery disease on all-cause mortality, myocardial infarction (MI), and ischemic stroke (IS) among hospitalized newly-diagnosed heart failure (HF) patients with left ventricular systolic dysfunction (LVSD).

**Methods:**

This retrospective cohort study included Medicare patients (aged ≥65 years) with ≥1 inpatient heart failure claim (index date = discharge date) during 01JAN2007-31DEC2013. Patients were required to have continuous enrollment for ≥1-year pre-index date (baseline: 1-year pre-index period) without a prior heart failure claim (in the 1 year pre-index prior to the index hospital admission); follow-up ran from the index date to death, disenrollment from the health plan, or the end of the study period, whichever occurred first. HF with LVSD patients, identified with diagnosis codes of systolic dysfunction (excluding baseline atrial fibrillation), were stratified based on prevalent coronary artery disease at baseline into coronary artery disease and non-coronary artery disease cohorts. Main outcomes were occurrence of major adverse cardiovascular events including all-cause mortality, myocardial infarction, and ischemic stroke. Propensity score matching (PSM) was used to balance patient characteristics. Kaplan-Meier curves of ACM and cumulative incidence distribution of MI/IS were presented.

**Results:**

Of 22,230 HF with LVSD patients, 15,827 (71.2%) had coronary artery disease and were overall more likely to be younger (79.8 vs 80.9 years), male (49.6% vs. 35.6%), white (86.2% vs 81.4%), with more prevalent comorbidities including hypertension (80.7% vs 74.3%), hyperlipidemia (67.7% vs 46.7%), and diabetes (46.3% vs 35.8%) (all *p* < 0.0001). After propensity score matching, cohorts included 5792 patients each. The coronary artery disease cohort had significantly higher cumulative incidence of myocardial infarction and ischemic stroke at the end of 7-year follow-up vs non-coronary artery disease (myocardial infarction = 50.0% vs 18.0%; ischemic stroke = 23.3% vs 18.7%; all p < 0.0001). Follow-up all-cause mortality rates were similar between the two cohorts.

**Conclusions:**

HF with LVSD patients with coronary artery disease had significantly higher incidence of ischemic stroke and myocardial infarction, but similar all-cause mortality compared to those without coronary artery disease.

**Electronic supplementary material:**

The online version of this article (10.1186/s12872-018-0991-1) contains supplementary material, which is available to authorized users.

## Highlights

- Our study assessed hospitalized heart failure with systolic dysfunction patients
- Among these patients, 71% had coronary artery disease
- Incidence of myocardial infarction was higher in those with coronary artery disease
- Coronary artery disease is also associated with higher incidence of ischemic stroke
- Mortality was similar between patients with and without coronary artery disease

## Background

Coronary artery disease (CAD) is the most common cause of heart failure (HF) and remains the primary cause of death, particularly in developed countries [1]. Approximately two-thirds of HF cases are attributable to underlying CAD [1]. HF is a chronic progressive disease that affected ~ 6 million people in the United States in 2012 [2]. Due to the aging of the US population and increased life expectancy, prevalence of HF is expected to increase to approximately 46% by 2030, resulting in > 8 million adults with HF [3]. HF due to left ventricular systolic dysfunction (LVSD), accounts for nearly half of HF cases [4]. Common risk factors for HF, such as hypertension and diabetes, also promote the development of atherosclerosis leading to CAD [5]. CAD accelerates the progression of HF with LVSD, leading to poor prognosis and a substantial increase in morbidity and mortality [6]. Given the increased burden of CAD in HF patients and the fact that CAD may have important therapeutic implications, the Heart Failure Society of America recommends testing for CAD in HF patients [1, 7]. Despite continuous improvements in HF management, morbidity and mortality remain unacceptably high: 22% of patients die within 1 year, and ~ 50% patients die within 5 years [3, 8]. The concomitant presence of CAD in HF patients has been reported to elevate the risk for cardiovascular (CV) outcomes including myocardial infarction (MI), IS, arrhythmia, mortality, and hospitalizations [9]. However, limited data evaluating the real world incidence of CV-related outcomes among hospitalized newly-diagnosed HF with LVSD patients with CAD and without AF is available. Additionally, HF patients without an atrial fibrillation (AF) diagnosis remain at considerably high risk for CV outcomes [10].

## Methods

This was a longitudinal, retrospective cohort study using a 5% random sample of the US Medicare database, including patients aged ≥65 years from 01JAN2006-31DEC2013. Medicare provides health insurance coverage to ~ 42 million persons aged ≥65 years as well as nearly 9 million persons aged < 65 years with end-stage kidney disease or a disability. For each beneficiary, claims from all settings of care were linked to create a longitudinal record of their health encounters, diagnoses, and drug prescriptions. No patient identity or medical records were disclosed for the purposes of this study. Since the data used for this study were de-identified and only aggregate results were reported, the study was approved by the Institutional Review Board as exempt. Compliance with all applicable laws and the Health Insurance Portability and Accountability Act (HIPAA) regulations were maintained.

**Fig. 1:**
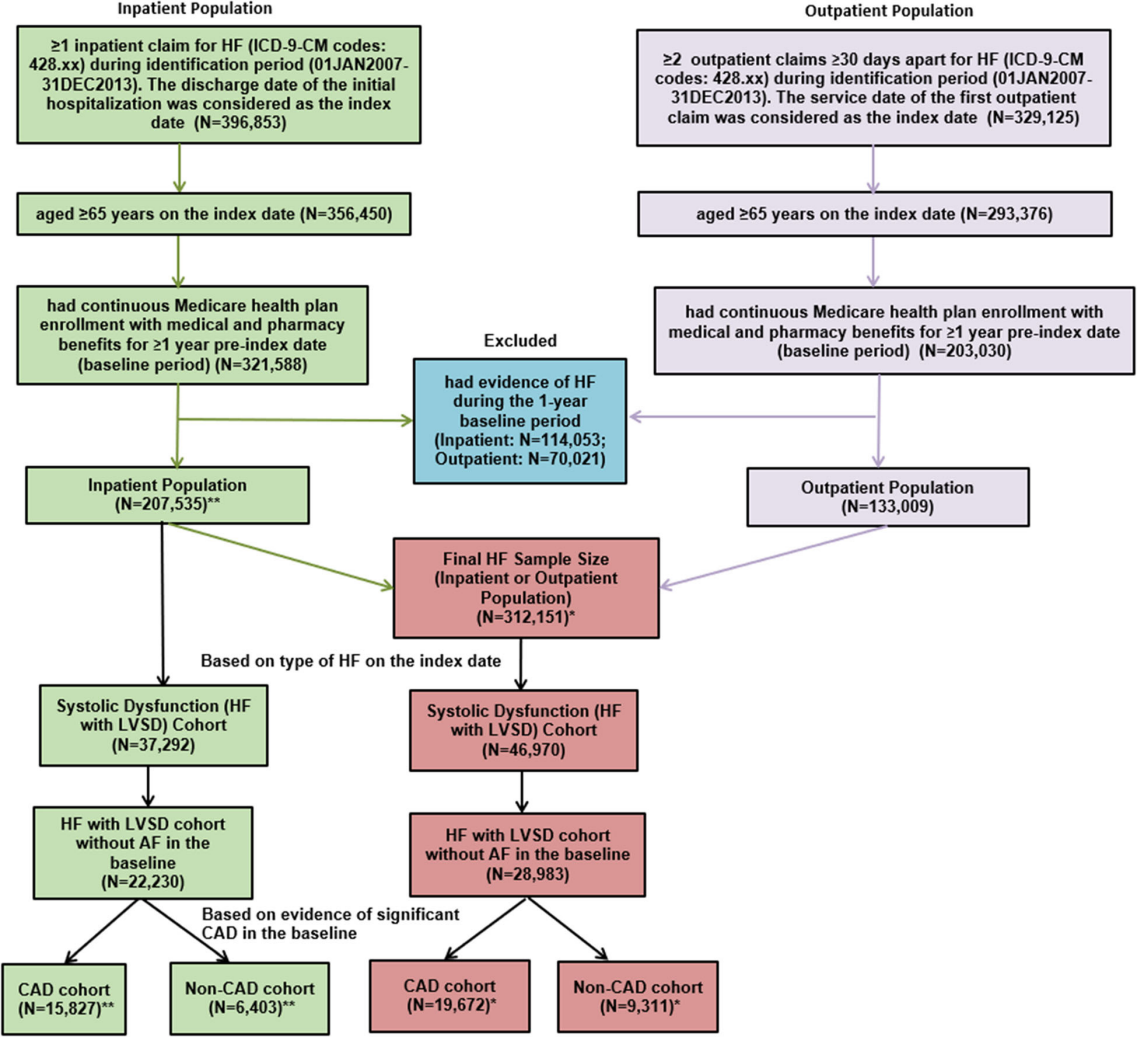
Patient Selection Criteria. AF: atrial fibrillation; CAD: coronary artery disease; HF: heart failure; LVSD: HF patients with left ventricular systolic dysfunction. *The final HF sample size used for the sensitivity analysis is less than the sum of the inpatient and outpatient populations because the populations were not mutually exclusive. **The HF sample used for main analysis

Patients were included in the study if they had ≥1 inpatient claim for HF (International classification of Diseases, 9th Revision, Clinical Modification [ICD-9-CM] code 428.xx) during the identification period (01JAN2007-31DEC2013); the discharge date of the first hospitalization was designated as the index date. Additionally, patients were required to have continuous enrollment in their Medicare health plan with medical and pharmacy benefits for ≥1-year pre-index date (baseline period: 1-year pre-index period including the index hospital admission). The follow-up period included the period after the index date until death, disenrollment from health plan, or end of the study period, whichever occurred first. Patients with evidence of HF during the 1-year period prior to the index hospital admission were excluded (Fig. 1).

HF patients with LVSD (ICD-9 CM codes 428.1, 428.20–428.23, 428.40–428.43) on the index date and without evidence of AF (ICD-9-CM code 427.31) in the baseline period (including the index hospital admission) were further stratified based on evidence of significant CAD in the baseline (including the index hospital admission) into CAD and non-CAD cohorts. We have included hospitalized HF patients without atrial fibrillation (AF) given the limited evidence on the burden of clinical outcomes as well as the conflicting evidence on the benefit of anticoagulation use in these patients [4]. Evidence of CAD was defined as having previously documented CAD (ICD-9-CM codes 410.x-414.x, 429.2, V45.81), history of prior coronary artery bypass graft (Current Procedural Terminology [CPT] codes 33,510–33,536 or ICD-9 CM procedure codes 36.10–36.17, 36.19), or history of percutaneous coronary intervention with or without stent (CPT codes 92,980–92,996 or ICD-9-CM codes 00.66, 36.01–36.09). Throughout the manuscript, included hospitalized HF patients with LVSD and without AF will be referred to as “HF with LVSD patients.” Additionally, newly-diagnosed HF patients identified with an inpatient or outpatient claim were included in a sensitivity analysis to see if there existed any differences in this population from the hospitalized HF population given the fact that the study included elderly patients aged ≥65 years.

### Baseline measures

Patient demographics including age, sex, race, and US geographic region as of the index date were assessed. Clinical characteristics including Charlson comorbidity index (CCI) score, CHADS_2_ score (congestive HF, hypertension, age ≥ 75 years, diabetes mellitus, prior stroke, or transient ischemic attack [TIA]), comorbidities (hypertension, hyperlipidemia, arrhythmia, anemia, diabetes, trauma, chronic renal insufficiency, malignant neoplasm, pneumonia, peripheral artery disease, anasarca, chronic obstructive pulmonary disease, dementia, hepatic disease, rheumatoid arthritis, depression, coagulation defect, obesity, varicose veins, thrombophilia, inflammatory bowel disease, arterial embolic events, peptic ulcer, alcohol abuse, pulmonary edema, bleeding diathesis), and prior clinical events (IS, TIA, venous thromboembolism [VTE], major bleeding) during the baseline period were assessed.

### Outcome measures

Main outcomes were occurrence of major adverse cardiovascular events including ACM, MI (ICD-9-CM codes: 410,412) and IS (ICD-9-CM codes: 433.01, 433.11, 433.21, 433.31, 433.81, 433.91, 434.01, 434.11, 434.91, 436). ACM rates, and cumulative incidence of MI and IS with death as a competing risk, were estimated by 60-day intervals during the first year of follow-up and by each year during the entire follow-up period (maximum 7 years) among newly-diagnosed “HF with LVSD patients” in the CAD versus non-CAD cohorts.

### Statistical analysis

Descriptive statistics (means and standard deviations for continuous variables, numbers and percentages for dichotomous/polychotomous variables) were provided for all study variables, including baseline demographic and clinical characteristics in the CAD and non-CAD cohorts. Statistical tests of significance (chi-square for categorical variables and t-test for continuous variables) were conducted to assess differences between the cohorts. Propensity Score Matching (PSM) was used to achieve baseline balance for patient characteristics. The propensity score was calculated via a logistic regression model, and the covariates adjusted in the model included all demographics, CCI score, CHADS_2_ score, comorbidities, and prior baseline clinical events. Each CAD patient was matched to a non-CAD patient within 0.01 units of the propensity score. The adequacy of the matching procedure was assessed by standardized difference for each of the matching variables; a difference of < 10% is considered well balanced [11]. Between the CAD and non-CAD cohorts, Kaplan-Meier (KM) curves of ACM were compared using the log-rank test; cumulative incidence distribution for MI and IS was compared using Gray’s test [12, 13]. All analyses were conducted using SAS® statistical software (Version 9.3, SAS Institute, Cary, North Carolina, 2012).

## Results

A total of 312,151 newly-diagnosed HF patients with either an inpatient or outpatient claim were identified, which included 207,535 hospitalized HF patients. These hospitalized HF patients comprised the study sample for the main analysis. Among them, 22,230 (10.7%) were diagnosed with LVSD on the index date and had no evidence of AF in the baseline period. Among newly-diagnosed hospitalized HF with LVSD patients, 15,827 (71.2%) were included in the CAD cohort and 6403 (28.8%) were included in the non-CAD cohort (Fig. 1).

### Baseline demographics and clinical characteristics

HF with LVSD patients in the CAD cohort were younger (79.8 vs 80.9 years) and were more frequently male (49.6% vs 35.6%) and white (86.2% vs 81.4%). Additionally, HF with LVSD patients in the CAD cohort had higher mean CCI scores (4.4 vs 3.5), CHADS_2_ scores (3.3 vs 3.0), and a higher percentage of comorbid hypertension (80.7% vs 74.3%), hyperlipidemia (67.7% vs 46.7%), diabetes (46.3% vs 35.8%), chronic renal insufficiency (32.1% vs 27.1%), arrhythmia (30.4% vs 23.2%), and peripheral artery disease (23.1% vs 13.0%). Also, the CAD cohort had a higher proportion of HF with LVSD patients diagnosed with IS (17.0% vs 10.9%) and TIA (3.9% vs 3.0%), but a lower proportion of patients diagnosed with VTE (5.8% vs 8.4%) in the baseline period (Table 1).

**Table 1:** Baseline Demographic and Clinical Characteristics of HF with LVSD Patients with and without CAD

Baseline Characteristics of HF Patients with LVSD	Before PSM						After 1:1 PSM					
	CAD Cohort (*N* = 15,827 )		Non-CAD Cohort (*N* = 6,403)				CAD Cohort (*N* = 5,792 )		Non-CAD Cohort (*N* = 5,792)			
	N/Mean	%/SD	N/Mean	%/SD	*p*-value	Std	N/Mean	%/SD	N/Mean	%/SD	*p*-value	Std
Age (Mean)	79.8	8.3	80.9	8.7	<.0001	13.0	80.8	8.5	80.7	8.6	0.4521	1.4
Age Group												
65-74	4,849	30.6%	1,714	26.8%	<.0001	8.6	1,566	27.0%	1,573	27.2%	0.8837	0.3
75-84	6,068	38.3%	2,296	35.9%	0.0005	5.1	2,123	36.7%	2,113	36.5%	0.8470	0.4
85+	4,910	31.0%	2,393	37.4%	<.0001	13.4	2,103	36.3%	2,106	36.4%	0.9538	0.1
Gender												
Male	7,852	49.6%	2,278	35.6%	<.0001	28.7	2,200	38.0%	2,174	37.5%	0.6183	0.9
Female	7,975	50.4%	4,125	64.4%	<.0001	28.7	3,592	62.0%	3,618	62.5%	0.6183	0.9
Race/Ethnicity												
White	13,636	86.2%	5,212	81.4%	<.0001	12.9	4,802	82.9%	4,787	82.6%	0.7120	0.7
Black	1,436	9.1%	924	14.4%	<.0001	16.7	755	13.0%	760	13.1%	0.8904	0.3
Hispanic	145	0.9%	57	0.9%	0.8535	0.3	44	0.8%	50	0.9%	0.5344	1.2
Asian	206	1.3%	65	1.0%	0.0780	2.7	51	0.9%	63	1.1%	0.2587	2.1
Native American	285	1.8%	110	1.7%	0.6723	0.6	112	1.9%	101	1.7%	0.4468	1.4
Other	92	0.6%	23	0.4%	0.0366	3.2	22	0.4%	23	0.4%	0.8813	0.3
Unknown	27	0.2%	12	0.2%	0.7861	0.4	6	0.1%	8	0.1%	0.5928	1.0
US Geographic Region												
Northeast	3,219	20.3%	1,271	19.9%	0.4113	1.2	1,170	20.2%	1,165	20.1%	0.9078	0.2
Midwest	4,149	26.2%	1,641	25.6%	0.3673	1.3	1,459	25.2%	1,491	25.7%	0.4950	1.3
South	6,370	40.2%	2,506	39.1%	0.1260	2.3	2,265	39.1%	2,284	39.4%	0.7177	0.7
West	2,051	13.0%	970	15.1%	<.0001	6.3	882	15.2%	838	14.5%	0.2503	2.1
Other	38	0.2%	15	0.2%	0.9357	0.1	16	0.3%	14	0.2%	0.7146	0.7
Comorbidity Indices												
Charlson Comorbidity Index	4.4	2.5	3.5	2.5	<.0001	34.5	3.7	2.1	3.7	2.6	0.1720	2.5
CHADS_2_ Score	3.3	1.2	3.0	1.1	<.0001	26.8	3.1	1.1	3.1	1.1	0.5115	1.2
Comorbid Conditions												
Hypertension	12,772	80.7%	4,756	74.3%	<.0001	15.4	4,397	75.9%	4,384	75.7%	0.7779	0.5
Hyperlipidemia	10,716	67.7%	2,993	46.7%	<.0001	43.4	2,896	50.0%	2,937	50.7%	0.4461	1.4
Anemia	7,298	46.1%	2,862	44.7%	0.0554	2.8	2,614	45.1%	2,607	45.0%	0.8960	0.2
Diabetes	7,320	46.3%	2,292	35.8%	<.0001	21.4	2,201	38.0%	2,179	37.6%	0.6734	0.8
Chronic Renal Insufficiency	5,078	32.1%	1,738	27.1%	<.0001	10.8	1,670	28.8%	1,632	28.2%	0.4342	1.5
Arrhythmia	4,804	30.4%	1,487	23.2%	<.0001	16.2	1,435	24.8%	1,424	24.6%	0.8126	0.4
Pneumonia	3,994	25.2%	1,864	29.1%	<.0001	8.7	1,707	29.5%	1,651	28.5%	0.2515	2.1
Trauma	4,064	25.7%	1,734	27.1%	0.0309	3.2	1,596	27.6%	1,563	27.0%	0.4912	1.3
Malignant Neoplasm	3,533	22.3%	1,404	21.9%	0.5207	1.0	1,313	22.7%	1,273	22.0%	0.3721	1.7
Peripheral Arterial Disease	3,663	23.1%	834	13.0%	<.0001	26.5	783	13.5%	814	14.1%	0.4035	1.6
Chronic Obstructive Pulmonary Disease	2,576	16.3%	1,056	16.5%	0.6929	0.6	968	16.7%	962	16.6%	0.8811	0.3
Dementia	1,725	10.9%	884	13.8%	<.0001	8.8	770	13.3%	768	13.3%	0.9563	0.1
Anasarca	1,666	10.5%	899	14.0%	<.0001	10.7	738	12.7%	757	13.1%	0.5985	1.0
Prior Ischemic Stroke	2,685	17.0%	696	10.9%	<.0001	17.7	701	12.1%	673	11.6%	0.4210	1.5
Prior Transient Ischemic Attack	625	3.9%	192	3.0%	0.0006	5.2	175	3.0%	183	3.2%	0.6676	0.8
Prior Venous Thromboembolism	912	5.8%	535	8.4%	<.0001	10.1	436	7.5%	429	7.4%	0.8046	0.5
Prior Major Bleeding*	597	3.8%	272	4.2%	0.0973	2.4	225	3.9%	239	4.1%	0.5071	1.2
Commander Criteria												
Documented previous CAD	15,787	99.75%	0	0.00%	N/A		5,775	99.71%	0	0.00%	N/A	
History of prior Coronary Artery Bypass Graft (CABG)	933	5.89%	0	0.00%			273	4.71%	0	0.00%		
History of percutaneous coronary intervention (PCI) with or without stent	2,430	15.35%	0	0.00%			730	12.60%	0	0.00%		

CAD: coronary artery disease; CHADS_2_: congestive HF, age ≥75 years, diabetes mellitus, prior stroke, transient ischemic attack or venous thromboembolism; HF: heart failure; PSM: propensity score matching; SD: standard deviation; STD: standardized difference

^*^Major bleeding was identified using the ICD-9-CM codes for intracranial Hemorrhage (ICD-9-CM: 430, 431, 432.0, 432.1, 432.9, 852.0x, 852.2x, 852.4x, 853.0), and extracranial hemorrhage (ICD-9-CM: 423.0, 455.2, 455.5, 455.8, 456.0, 456.20, 459.0, 530.7, 530.82, 531.0-531.6, 532.0-532.6, 533.0-533.6, 534.0-534.6, 535.01-535.61, 537.83, 562.02, 562.03, 562.12, 562.13, 568.81, 569.3, 569.85, 578.0, 578.1, 578.9, 593.81,599.7, 719.11, 784.7, 784.8, and 786.3)

#### Post-propensity score matching results

After 1:1 PSM, each cohort included 5792 patients. The cohorts were well-balanced based on baseline demographic and clinical characteristics, with a standardized difference of < 10% after matching (Table 1). The median follow-up period was 15.7 months in both cohorts.

### ACM, MI, and IS rates among HF patients with and without CAD in the entire follow-up period after PSM

#### All-cause mortality rate

**Fig. 2:**
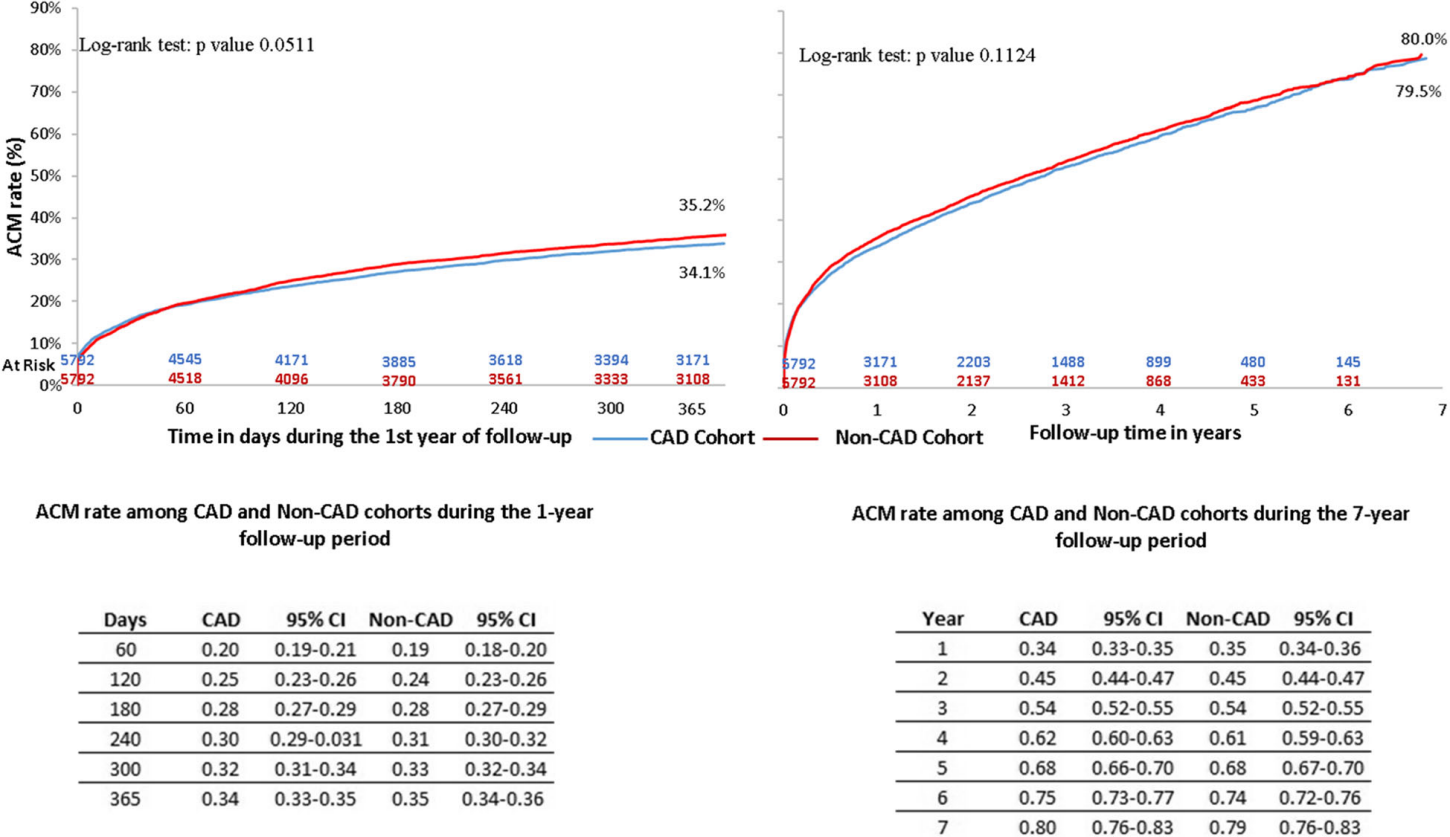
Follow-up ACM Rates among CAD vs Non-CAD Patients - after PSM. ACM: all-cause mortality; CAD: coronary artery disease; CI: confidence interval; PSM: propensity score matching

The ACM rate in the CAD cohort was 34% at the end of 1 year, followed by 54 and 68% at the end of years 3 and 5, respectively (Fig. 2). The ACM rate did not significantly differ between the CAD and non-CAD cohorts (34.1% vs 35.2%, *p* = 0.0511) during the first year of follow-up nor during the entire duration of follow-up (80.0% vs 79.5%, *p* = 0.1124).

#### Cumulative incidence of myocardial infarction with death as competing risk

**Fig. 3:**
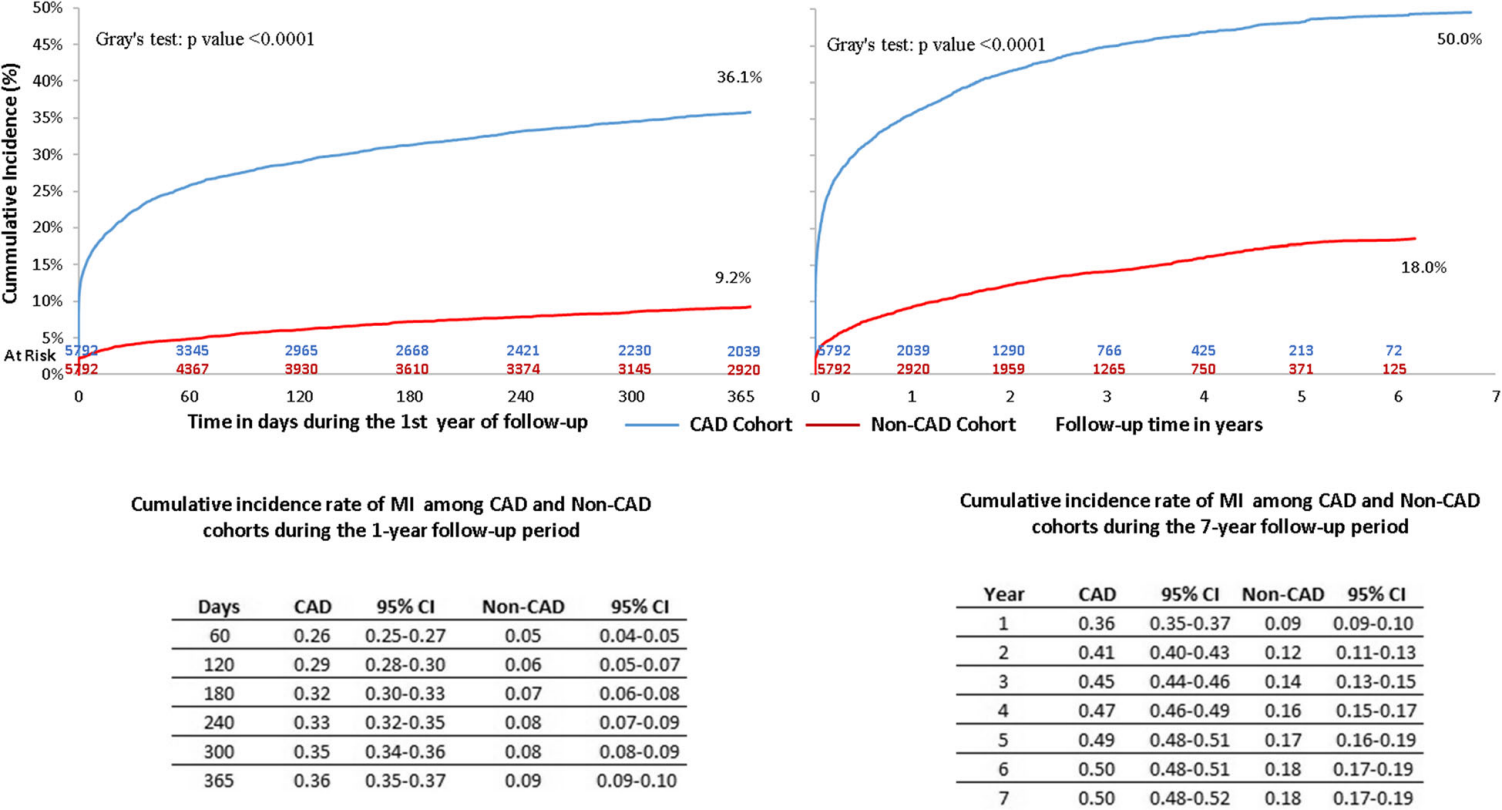
Follow-up Cumulative Incidence of MI among CAD vs Non-CAD Patients – after PSM. CAD: coronary artery disease; CI: confidence interval; MI: myocardial infarction; PSM: propensity score matching

The cumulative incidence rate of MI in the CAD cohort was 36% at the end of 1 year, followed by 45, and 49% at the end of years 3 and 5, respectively (Fig. 3). The CAD cohort had a significantly higher cumulative incidence of MI during the first year of follow-up (36.1% vs 9.2%, *p* < 0.0001), as well as a significantly higher cumulative incidence rate of MI (50.0% vs 18.0%, *p* < 0.0001) during the entire follow-up period.

#### Cumulative incidence of ischemic stroke with death as competing risk

**Fig. 4:**
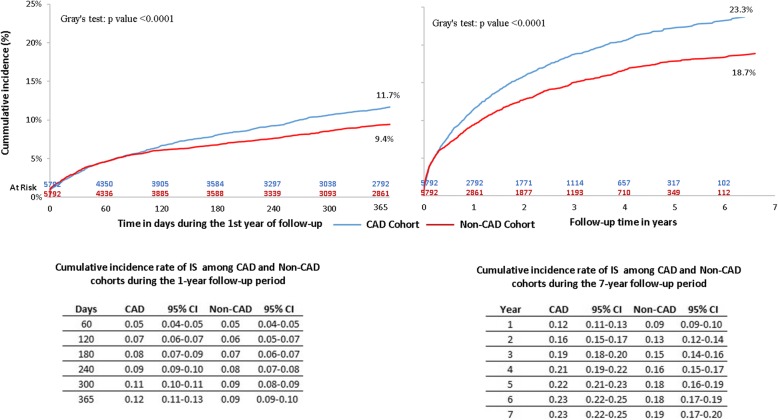
Follow-up Cumulative Incidence of IS among CAD vs Non-CAD Patients – after PSM CAD: coronary artery disease; CI: confidence interval; IS: ischemic stroke; PSM: propensity score matching

The cumulative incidence rate of IS in the CAD cohort was 12% at the end of 1 year, followed by 19 and 22% at the end of years 3 and 5, respectively (Fig. 4). As was observed with MI, the CAD cohort had a significantly higher cumulative incidence of IS during the first year (11.7% vs 9.4%, *p* < 0.0001) and the entire follow-up period (23.3% vs 18.7%, p < 0.0001).

### Sensitivity analysis

A total of 312,151 newly-diagnosed HF patients including either inpatient (*N* = 207,535) or outpatient (*N* = 133,009) claims were included for sensitivity analysis, of which 28,983 (9.3%) were diagnosed with LVSD on the index date and had no evidence of AF in the baseline period. Among these HF with LVSD patients, 19,672 (67.9%) were included in the CAD cohort and 9311 (32.1%) were included in the non-CAD cohort (Fig. 1). After 1:1 PSM, each cohort included 8069 patients with a median follow-up period of 22 months (Additional file 1: Table S1).

The ACM rate did not significantly differ between the cohorts (27.1% vs 26.6%, *p* = 0.2153) during the first year of the follow-up period; however, the CAD cohort had a significantly higher ACM rate (70.6% vs 66.5%, *p* = 0.0069) during the entire follow-up period. The cumulative incidence rate of MI in both cohorts is consistent with that in the primary analysis, suggesting that the risk of MI is nearly four times higher during the first year and three times higher at the end of the follow-up period in the CAD cohort as compared to the non-CAD cohort. The CAD cohort had a significantly higher cumulative incidence of MI (33.3% vs 8.5%, *p* < 0.0001) during the first year and during the entire follow-up period (49.5% vs 18.7%, *p* < 0.0001). The cumulative incidence of IS did not significantly differ between the cohorts during the first year of follow-up (12.0% vs 11.0%, *p* = 0.0633); however, patients in the CAD cohort had a significantly higher cumulative incidence of IS (25.9% vs 23.2%, *p* = 0.0005) during the complete follow-up period (data not shown).

## Discussion

To the best of our knowledge, this is the first real-world study to evaluate CV-related outcomes among HF with LVSD patients with concomitant CAD and without AF. Despite evidence in the literature regarding CV condition-related outcomes among HF and CAD populations separately, limited real-world evidence is available among patients diagnosed with concomitant HF and CAD, with LVSD and without AF in United States [9]. In this large cohort of hospitalized newly-diagnosed HF with LVSD patients without AF, patients with CAD were observed to have a significantly higher incidence of IS and MI and did not significantly differ in ACM rates compared to those without CAD. CAD is considered as a major cause of HF with LVSD and is prevalent in about two-thirds of the patients with HF with LVSD [14]. Additionally, CAD has been shown to adversely affect the prognosis in HF patients; the results of the current study help us to understand the impact of documented CAD among HF with LVSD patients. The results also provide evidence on stroke risk among HF with LVSD patients without AF, thereby allowing health care providers to enhance treatment planning among these hospitalized newly-diagnosed HF with LVSD patients without AF and with CAD.

The results of our study showed that the ACM rates did not significantly differ between the hospitalized HF with LVSD patients with and without CAD, probably due to the older age of the patients in both cohorts. Elderly patients have higher prevalence of multiple comorbidities, all of which complicate patient management thereby increasing the mortality rates [15, 16]. Among Medicare beneficiaries, two-thirds have multiple chronic conditions, with prevalence ranging from 62% in those aged 65–74 years, 75.7% in those aged 75–84 years, and 81.5% in those aged ≥85 years. However, the results of our sensitivity analysis in the total HF with LVSD patients showed that CAD cohort had significantly higher ACM rates than those without CAD in the entire follow-up period. Previous studies observed CAD as a significant predictor of mortality among HF patients with a hazard ratio ranging from 1 to 3.4 [17, 18]. Additionally, the results of our study showed that the 1-year mortality in this elderly HF population was ~ 35% in both cohorts, which is consistent with a previous study conducted by Hernandez et al., which observed a 32% mortality rate in older HF patients without AF [19]. These results highlight the poor prognosis and low survival rates among HF patients. Notably, the 1-year mortality rate in HF patients is high and comparable to that of cancer patients (~ 42%) [20]. Additionally, the given that about 20% of the patients had evidence of malignant neoplasm in the baseline period, the ACM rate in our study should be interpreted with caution as the cause of death cannot be attributed to either coronary events or heart failure.

The results of our study showed that among patients diagnosed with CAD, the cumulative incidence of MI was approximately four times higher for the 1-year post-discharge period and three times higher during the entire follow-up period as compared to HF with LVSD patients without CAD. The results indicate that the impact of CAD on follow-up MI was larger in the first year after HF diagnosis and declined a bit in later years but remained significantly higher over time. In a study conducted by Rusinaru et al., which evaluated 10-year outcomes among HF patients with CAD, the risk of MI was approximately 2.4 times higher in HF patients with CAD as compared to those without CAD [17]. Despite the differences in the study design and sample selection criteria, these studies support the findings in the current study, concluding that CAD is associated with higher risk of MI among HF patients. These results highlight the importance of developing an appropriate treatment strategy that may reduce the risk of MI in HF patients. Rivaroxaban 2.5 mg and 5 mg (twice-daily) has been shown to reduce the risk of composite efficacy endpoint (ACM, MI, stroke) among HF patients in the ATLAS-2 trial [21]. Additionally, the results of an unpublished subgroup analysis of ATLAS-2, which included clinically diagnosed HF patients at the time of their acute coronary syndrome event, showed that rivaroxaban 2.5 mg (twice-daily) reduced the risk of MI or stroke to 11.6% as compared to 18.6% in placebo [21]. Although these results suggest that treatment with rivaroxaban could be beneficial in improving overall health and thereby reduce the health care burden in HF patients, the results of a recent large landmark trial found that rivaroxaban 2.5 mg (twice daily) did not show a significant improvement for cardiovascular outcomes including death, MI, or IS among patients hospitalized with HF and reduced ejection fraction and with CAD and no AF [22]. The authors mentioned that the most likely reason for this could be that the thrombin-mediated events were not the significant drivers of HF-related events in recently hospitalized HF patients [22].

The results of our study showed that the incidence rate of IS was significantly higher in hospitalized HF with LVSD patients with CAD as compared to those without CAD. Evidence from the literature suggests that the incidence rate of stroke among HF patients with and without AF is approximately 0.85 and 0.69 per 100 person years, respectively [23]. Additionally, in a study conducted by Melgaard et al., the crude relative risk of IS was about 2 times higher in HF patients without AF as compared to those with AF [24]. These results highlight the considerably high risk of stroke in HF patients without AF, and the potential importance of anticoagulation in these HF patients with sinus rhythm. In a comprehensive review on the effectiveness of warfarin in reducing the risk of stroke in HF patients without AF, it was observed that warfarin had no convincing evidence of reducing mortality or vascular events [25]. Although warfarin use has shown some beneficial effect to reduce IS, the medication was associated with serious adverse events including increased risk of bleeding [25]. The efficacy of novel oral anticoagulants (rivaroxaban) on ACM, MI, and stroke among hospitalized HF patients without AF and with CAD is currently being investigated in the ongoing COMMANDER-HF trial [21]. Additionally, estimating the risk of stroke and mortality in HF patients provides an important tool to identify subset(s) of patients that may benefit from thromboprophylaxis [10].

In a sensitivity analysis of inpatient and outpatient HF with LVSD claims, the results remained consistent and support the robust finding of higher risk of MI in patients in the CAD cohort as compared to those in the non-CAD cohort during the 1-year and long-term follow-up periods. Despite the similar ACM rates between the two cohorts in the main analysis, the results of the sensitivity analysis showed that CAD is associated with a higher risk of ACM in HF with LVSD patients, which is consistent with the previous studies. Additionally, in contrast to the main analysis, the results of the sensitivity analysis showed that the incidence of IS was not significantly different in both the CAD and non-CAD cohorts during the 1-year follow-up period, although it is numerically higher in the CAD cohort. Therefore, CAD qualifies as a viable candidate for sub-classification among HF with LVSD patients due to the condition’s high prevalence and associated burden, irrespective of the patient being hospitalized. Further research will help to identify high-risk HF patients and provide efficient treatment, thus reducing the clinical and economic burden of the disease.

The findings from our study should be viewed in the context of some study limitations. Our study relied on retrospective claims data. While claims data are extremely valuable for the efficient and effective examination of health care outcomes, treatment patterns, and costs, they are collected for business purposes and not with research intentions. Additionally, incorrect coding may have resulted in misclassification. For example, the presence of a diagnosis code on medical claims may not indicate a positive presence of a disease and may be incorrectly coded or included as rule-out criteria rather than indicating the actual disease. Also, certain clinical and disease-specific parameters are not readily available in claims data including smoking status and HF severity, which may have influenced study outcomes. Furthermore, the study relied on diagnosis codes to define LVSD, resulting in a relatively smaller HF with LVSD population due to inadequate coding in the claims data. Conversely, using diagnosis codes to identify the HF with LVSD population may have resulted in higher sensitivity, while specificity is not certain as many people who had LVSD were not coded as such. Similarly, as AF in the baseline period was identified using diagnosis codes, it is possible that patients with AF were still included in the study. Further, it could be possible that AF occurred later during the post-index period, which was not captured in our study. The requirement of no HF diagnosis in the 12-month baseline period in our study may have failed to identify “true newly-diagnosed HF patients” as it is not necessary that all HF patients have annual health care visits; thus, many chronic HF patients may have been included in the study. HF is a heterogeneous condition, and this analysis does not distinguish between HF etiologies. Considering the fact that MI is a potent risk factor for HF, the results of our study could be biased and should be interpreted with caution as it is difficult to determine whether MI has caused HF or if HF caused MI. Further analysis is needed to better understand differences in the burden of disease in these patient groups. The current study represented only US data from a specific subpopulation (Medicare enrollees) who were mostly elderly patients. Therefore, the general applicability of our findings to younger patients requires further study.

## Conclusions

HF with LVSD patients with CAD and without AF were at a substantially higher burden of MI and IS within the first year of diagnosis, resulting in a poor prognosis. The impact of CAD on CV-related outcomes is substantial, with a higher burden of MI and IS during the entire follow-up period. Although HF with LVSD patients with CAD had a similar ACM rate as those without, mortality was high, warranting optional treatment to improve patient outcomes. Secondary prevention and appropriate management of CAD for this high-risk population may further reduce HF-associated burden.

## Additional file

Additional file 1: Baseline Demographic and Clinical Characteristics Before and After Propensity Score Matching in the Inpatient or Outpatient HF Patients (Sensitivity Analysis). *Major bleeding was identified using the ICD-9-CM codes for intracranial Hemorrhage (ICD-9-CM: 430, 431, 432.0, 432.1, 432.9, 852.0x, 852.2x, 852.4x, 853.0), and extracranial hemorrhage (ICD-9-CM: 423.0, 455.2, 455.5, 455.8, 456.0, 456.20, 459.0, 530.7, 530.82, 531.0-531.6, 532.0-532.6, 533.0-533.6, 534.0-534.6, 535.01-535.61, 537.83, 562.02, 562.03, 562.12, 562.13, 568.81, 569.3, 569.85, 578.0, 578.1, 578.9, 593.81,599.7, 719.11, 784.7, 784.8, and 786.3). (DOCX 33 kb)

## Abbreviations

ACM: All-cause mortality
CAD: Coronary artery disease
CCI: Charlson comorbidity index
CPT: Current procedural terminology
CV: Cardiovascular
HF: Heart failure
IS: Ischemic stroke
LVSD: Left ventricular systolic dysfunction
MI: Myocardial infarction
PSM: Propensity score matching
SD: Systolic dysfunction
TIA: Transient ischemic attack
VTE: Venous thromboembolism
