# Supplementary material for: Cardiovascular outcomes among elderly patients with heart failure and coronary artery disease and without atrial fibrillation: a retrospective cohort study
Source: BMC Cardiovasc Disord. 2019 Jan 15;19:19. doi: 10.1186/s12872-018-0991-1 (PMC6334438; doi:10.1186/s12872-018-0991-1)
Supplement: Supplementary file 1 — Baseline Demographic and Clinical Characteristics Before and After Propensity Score Matching in the Inpatient or Outpatient HF Patients (Sensitivity Analysis). *Major bleeding was identified using the ICD-9-CM codes for intracranial Hemorrhage (ICD-9-CM: 430, 431, 432.0, 432.1, 432.9, 852.0x, 852.2x, 852.4x, 853.0), and extracranial hemorrhage (ICD-9-CM: 423.0, 455.2, 455.5, 455.8, 456.0, 456.20, 459.0, 530.7, 530.82, 531.0-531.6, 532.0-532.6, 533.0-533.6, 534.0-534.6, 535.01-535.61, 537.83, 562.02, 562.03, 562.12, 562.13, 568.81, 569.3, 569.85, 578.0, 578.1, 578.9, 593.81,599.7, 719.11, 784.7, 784.8, and 786.3). (DOCX 33 kb) [file 12872_2018_991_MOESM1_ESM.docx]

| **Baseline Demographic and Clinical Characteristics** | **Before PSM Matching** | | | | | | **After 1:1 PSM Matching** | | | | | |
| --- | --- | --- | --- | --- | --- | --- | --- | --- | --- | --- | --- | --- |
|  | **CAD Cohort** | | **Non-CAD Cohort** | |  |  | **CAD Cohort** | | **Non-CAD Cohort** | |  |  |
|  | **(N= 19,672 )** | | **(N= 9,311)** | |  |  | **(N=8,069 )** | | **(N= 8,069)** | |  |  |
|  | **N/Mean** | **%/SD** | **N/Mean** | **%/SD** | **p-value** | **Std** | **N/Mean** | **%/SD** | **N/Mean** | **%/SD** | **p-value** | **Std** |
| **Age (Mean,SD)** | 79.4 | 8.2 | 80.2 | 8.5 | <.0001 | 10.0 | 80.2 | 8.4 | 80.1 | 8.5 | 0.4066 | 1.3 |
| **Age Group** |  |  |  |  |  |  |  |  |  |  |  |  |
| 65-74 | 6,287 | 32.0% | 2,712 | 29.1% | <.0001 | 6.2 | 2,333 | 28.9% | 2,346 | 29.1% | 0.8216 | 0.4 |
| 75-84 | 7,672 | 39.0% | 3,450 | 37.1% | 0.0015 | 4.0 | 3,080 | 38.2% | 3,063 | 38.0% | 0.7828 | 0.4 |
| 85+ | 5,713 | 29.0% | 3,149 | 33.8% | <.0001 | 10.3 | 2,656 | 32.9% | 2,660 | 33.0% | 0.9466 | 0.1 |
| **Gender** |  |  |  |  |  |  |  |  |  |  |  |  |
| Male | 10,029 | 51.0% | 3,409 | 36.6% | <.0001 | 29.3 | 3,174 | 39.3% | 3,187 | 39.5% | 0.8341 | 0.3 |
| Female | 9,643 | 49.0% | 5,902 | 63.4% | <.0001 | 29.3 | 4,895 | 60.7% | 4,882 | 60.5% | 0.8341 | 0.3 |
| **Race/Ethnicity** |  |  |  |  |  |  |  |  |  |  |  |  |
| White | 16,991 | 86.4% | 7,656 | 82.2% | <.0001 | 11.4 | 6,726 | 83.4% | 6,731 | 83.4% | 0.9158 | 0.2 |
| Black | 1,740 | 8.8% | 1,223 | 13.1% | <.0001 | 13.7 | 957 | 11.9% | 959 | 11.9% | 0.9612 | 0.1 |
| Hispanic | 187 | 1.0% | 95 | 1.0% | 0.5724 | 0.7 | 78 | 1.0% | 81 | 1.0% | 0.8110 | 0.4 |
| Asian | 253 | 1.3% | 116 | 1.2% | 0.7753 | 0.4 | 122 | 1.5% | 102 | 1.3% | 0.1784 | 2.1 |
| Native American | 363 | 1.8% | 170 | 1.8% | 0.9083 | 0.1 | 150 | 1.9% | 151 | 1.9% | 0.9536 | 0.1 |
| Other | 106 | 0.5% | 34 | 0.4% | 0.0464 | 2.6 | 30 | 0.4% | 33 | 0.4% | 0.7049 | 0.6 |
| Unknown | 32 | 0.2% | 17 | 0.2% | 0.7000 | 0.5 | 6 | 0.1% | 12 | 0.1% | 0.1571 | 2.2 |
| **US Geographic Region** |  |  |  |  |  |  |  |  |  |  |  |  |
| Northeast | 4,039 | 20.5% | 1,898 | 20.4% | 0.7718 | 0.4 | 1,655 | 20.5% | 1,652 | 20.5% | 0.9533 | 0.1 |
| Midwest | 4,954 | 25.2% | 2,210 | 23.7% | 0.0076 | 3.4 | 1,921 | 23.8% | 1,935 | 24.0% | 0.7961 | 0.4 |
| South | 7,946 | 40.4% | 3,609 | 38.8% | 0.0081 | 3.3 | 3,187 | 39.5% | 3,181 | 39.4% | 0.9230 | 0.2 |
| West | 2,679 | 13.6% | 1,569 | 16.9% | <.0001 | 9.0 | 1,284 | 15.9% | 1,277 | 15.8% | 0.8801 | 0.2 |
| Other | 54 | 0.3% | 25 | 0.3% | 0.9271 | 0.1 | 22 | 0.3% | 24 | 0.3% | 0.7678 | 0.5 |
| **Comorbidity Indices** |  |  |  |  |  |  |  |  |  |  |  |  |
| Charlson Comorbidity Index (Mean,SD) | 4.0 | 2.5 | 3.0 | 2.5 | <.0001 | 41.7 | 3.2 | 2.2 | 3.2 | 2.5 | 0.1904 | 2.1 |
| CHADS_2_ Score (Mean,SD) | 3.1 | 1.3 | 2.7 | 1.2 | <.0001 | 35.4 | 2.8 | 1.2 | 2.8 | 1.2 | 0.0972 | 2.6 |
| **Comorbid Conditions** |  |  |  |  |  |  |  |  |  |  |  |  |
| Diabetes | 8,965 | 45.6% | 3,259 | 35.0% | <.0001 | 21.7 | 3,090 | 38.3% | 3,049 | 37.8% | 0.5062 | 1.0 |
| Hypertension | 15,660 | 79.6% | 6,734 | 72.3% | <.0001 | 17.1 | 6,047 | 74.9% | 6,052 | 75.0% | 0.9276 | 0.1 |
| Peripheral Arterial Disease | 4,432 | 22.5% | 1,160 | 12.5% | <.0001 | 26.7 | 1,101 | 13.6% | 1,109 | 13.7% | 0.8547 | 0.3 |
| Malignant Neoplasm | 4,467 | 22.7% | 2,069 | 22.2% | 0.3549 | 1.2 | 1,835 | 22.7% | 1,797 | 22.3% | 0.4738 | 1.1 |
| Arterial Embolic Events | 177 | 0.9% | 33 | 0.4% | <.0001 | 6.9 | 37 | 0.5% | 33 | 0.4% | 0.6318 | 0.8 |
| Dementia | 1,858 | 9.4% | 1,026 | 11.0% | <.0001 | 5.2 | 873 | 10.8% | 884 | 11.0% | 0.7810 | 0.4 |
| Anemia | 8,401 | 42.7% | 3,618 | 38.9% | <.0001 | 7.8 | 3,339 | 41.4% | 3,237 | 40.1% | 0.1022 | 2.6 |
| Pulmonary Edema | 151 | 0.8% | 49 | 0.5% | 0.0205 | 3.0 | 48 | 0.6% | 45 | 0.6% | 0.7551 | 0.5 |
| Anasarca | 2,058 | 10.5% | 1,326 | 14.2% | <.0001 | 11.5 | 1,063 | 13.2% | 1,048 | 13.0% | 0.7262 | 0.6 |
| Chronic Renal Insufficiency | 5,662 | 28.8% | 2,065 | 22.2% | <.0001 | 15.2 | 1,917 | 23.8% | 1,898 | 23.5% | 0.7248 | 0.6 |
| Hepatic Disease | 925 | 4.7% | 434 | 4.7% | 0.8776 | 0.2 | 398 | 4.9% | 379 | 4.7% | 0.4848 | 1.1 |
| Thrombophilia | 289 | 1.5% | 119 | 1.3% | 0.1974 | 1.6 | 112 | 1.4% | 110 | 1.4% | 0.8925 | 0.2 |
| Peptic Ulcer | 135 | 0.7% | 63 | 0.7% | 0.9259 | 0.1 | 58 | 0.7% | 55 | 0.7% | 0.7770 | 0.4 |
| Bleeding Diathesis | 14 | 0.1% | 4 | 0.0% | 0.3681 | 1.2 | 3 | 0.0% | 4 | 0.0% | 0.7054 | 0.6 |
| Chronic Obstructive Pulmonary Disease | 2,867 | 14.6% | 1,280 | 13.7% | 0.0605 | 2.4 | 1,161 | 14.4% | 1,137 | 14.1% | 0.5888 | 0.9 |
| Hyperlipidemia | 13,537 | 68.8% | 4,482 | 48.1% | <.0001 | 42.9 | 4,305 | 53.4% | 4,316 | 53.5% | 0.8622 | 0.3 |
| Depression | 674 | 3.4% | 337 | 3.6% | 0.4026 | 1.0 | 302 | 3.7% | 285 | 3.5% | 0.4747 | 1.1 |
| Obesity | 482 | 2.5% | 233 | 2.5% | 0.7889 | 0.3 | 200 | 2.5% | 204 | 2.5% | 0.8403 | 0.3 |
| Arrhythmia | 5,802 | 29.5% | 1,922 | 20.6% | <.0001 | 20.5 | 1,777 | 22.0% | 1,805 | 22.4% | 0.5958 | 0.8 |
| Pneumonia | 4,264 | 21.7% | 2,066 | 22.2% | 0.3233 | 1.2 | 1,856 | 23.0% | 1,834 | 22.7% | 0.6801 | 0.6 |
| Varicose Veins | 398 | 2.0% | 221 | 2.4% | 0.0540 | 2.4 | 165 | 2.0% | 171 | 2.1% | 0.7408 | 0.5 |
| Coagulation Defect | 437 | 2.2% | 191 | 2.1% | 0.3530 | 1.2 | 169 | 2.1% | 172 | 2.1% | 0.8696 | 0.3 |
| Rheumatoid Arthritis | 756 | 3.8% | 390 | 4.2% | 0.1586 | 1.8 | 328 | 4.1% | 332 | 4.1% | 0.8737 | 0.3 |
| Inflammatory Bowel Disease | 164 | 0.8% | 82 | 0.9% | 0.6838 | 0.5 | 63 | 0.8% | 68 | 0.8% | 0.6609 | 0.7 |
| Alcohol Abuse | 166 | 0.8% | 90 | 1.0% | 0.2969 | 1.3 | 79 | 1.0% | 76 | 0.9% | 0.8087 | 0.4 |
| Trauma | 4,854 | 24.7% | 2,316 | 24.9% | 0.7137 | 0.5 | 1,984 | 24.6% | 1,990 | 24.7% | 0.9127 | 0.2 |
| Prior ischemic Stroke | 3,319 | 16.9% | 934 | 10.0% | <.0001 | 20.1 | 882 | 10.9% | 905 | 11.2% | 0.5640 | 0.9 |
| Prior Transient Ischemic Attack | 773 | 3.9% | 287 | 3.1% | 0.0003 | 4.6 | 251 | 3.1% | 260 | 3.2% | 0.6858 | 0.6 |
| Prior Venous Thromboembolism | 1,062 | 5.4% | 633 | 6.8% | <.0001 | 5.9 | 506 | 6.3% | 507 | 6.3% | 0.9741 | 0.1 |
| Prior Major Bleeding | 670 | 3.4% | 304 | 3.3% | 0.5342 | 0.8 | 267 | 3.3% | 269 | 3.3% | 0.9300 | 0.1 |
| **Commander Criteria** |  |  |  |  |  |  |  |  |  |  |  |  |
| Documented previous CAD | 19,632 | 99.80% | 0 | 0.00% | N/A | | 8,045 | 99.70% | 0 | 0.00% | N/A | |
| History of prior Coronary Artery Bypass Graft (CABG) | 1,007 | 5.12% | 0 | 0.00% |  |  | 284 | 3.52% | 0 | 0.00% |  |  |
| History of percutaneous coronary intervention (PCI) with or without stent | 2,662 | 13.53% | 0 | 0.00% |  |  | 902 | 11.18% | 0 | 0.00% |  |  |
